# Protective effects of *Lagerstroemia speciosa* against paracetamol-induced renal and testicular toxicity in rats via antioxidant, anti-inflammatory, and anti-apoptotic mechanisms

**DOI:** 10.3389/ftox.2026.1751678

**Published:** 2026-03-04

**Authors:** Nagham E. Elsheshtawy, Engy F. Risha, Fatma M. Abdelhamid, Bodour S. Rajab, Rehab Bagadood, Bayan Bokhari, Ghadir Sindi, Ahmed I. Ateya, Shaymaa Rezk, Mohamed E. El-Boshy

**Affiliations:** 1 Clinical Pathology Department, Faculty of Veterinary Medicine, Mansoura University, Mansoura, Egypt; 2 Department of Laboratory Medicine, Faculty of Applied Medical Sciences, Umm Al-Qura University, Makkah, Saudi Arabia; 3 Animal Husbandry and Animal Wealth Development Department, Faculty of Veterinary Medicine, Mansoura University, Mansoura, Egypt; 4 Cytology and Histology Department, Faculty of Veterinary Medicine, Mansoura University, Mansoura, Egypt

**Keywords:** apoptosis, inflammation, Lagerstroemia speciosa, oxidative stress, paracetamol toxicity, renal injury, reproductive toxicity

## Abstract

**Introduction:**

Paracetamol (PCM) is widely used as an analgesic; however, at high doses, it is well recognized for its hepatotoxic effects and is increasingly associated with renal and reproductive damage.

**Methods:**

In the present study, the protective potential of ethanolic *Lagerstroemia speciosa* leaf extract (EELS; 500 mg/kg/day, orally for 24 days) was investigated against PCM-induced kidney and testicular injury in male rats, using N-acetyl cysteine (NAC) as a reference treatment.

**Results:**

PCM administration led to pronounced impairment in sperm quality and significant disturbances in serum biochemical parameters, reflected by elevated renal function markers, sodium, and phosphorus levels, together with reduced calcium, potassium, and testosterone. These changes were accompanied by clear evidence of oxidative stress, as indicated by increased malondialdehyde and decreased reduced glutathione. Moreover, inflammatory and apoptotic responses were markedly intensified, whereas antioxidant and steroidogenic regulators were suppressed. Histopathological findings further confirmed extensive structural damage in renal and testicular tissues. Notably, treatment with EELS or NAC substantially mitigated these alterations, restoring most parameters toward normal values.

**Conclusion:**

Collectively, these findings demonstrate that EELS exerts notable nephroprotective and gonadoprotective effects comparable to NAC, largely through the attenuation of oxidative stress, inflammation, and apoptosis.

## Graphical Abstract

**Figure:**
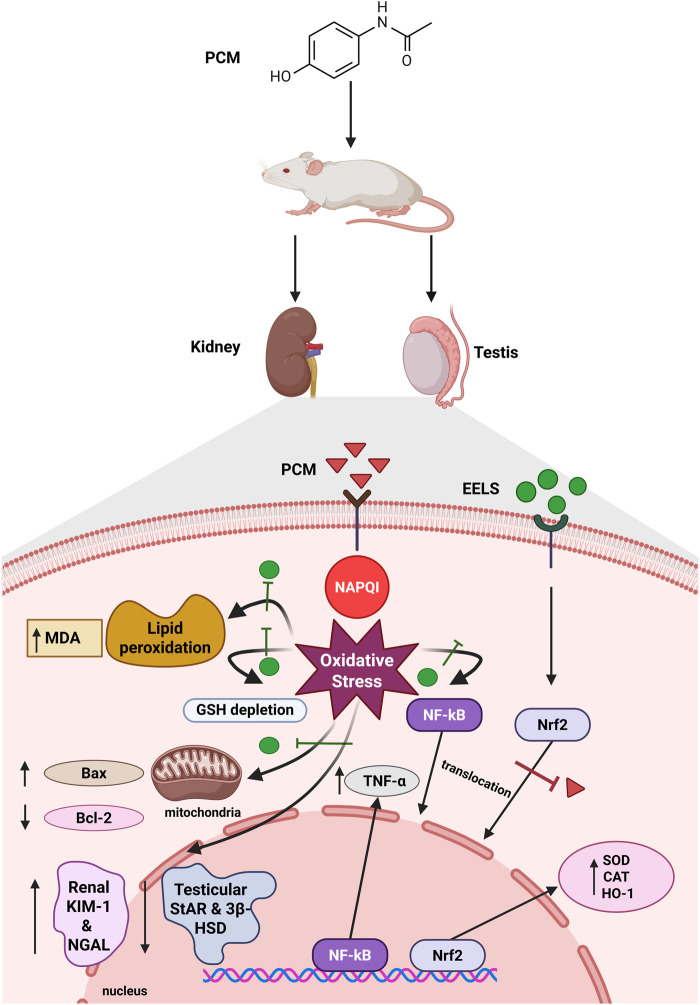

## 1 Introduction

Paracetamol (PCM) is a widely used antipyretic and analgesic drug that is considered safe at therapeutic doses (Elsafty et al., 2024). However, overdose is well known to induce acute hepatotoxicity as the predominant adverse outcome, while renal and testicular injuries, although documented, occur less frequently (Diab et al., 2020).

Hepatic biotransformation of PCM occurs mainly via glucuronidation and sulfation, with metabolites eliminated through renal excretion (Chowdhury et al., 2020). At toxic doses, PCM is converted into the highly reactive intermediate N-acetyl-p-benzoquinone imine (NAPQI), which exceeds the detoxification capacity of reduced glutathione (GSH) (Canayakin et al., 2016). GSH depletion results in excessive reactive oxygen species (ROS) generation, initiating lipid peroxidation and oxidative injury in extrahepatic organs, particularly the kidney and testes (Dua et al., 2022). Accordingly, oxidative stress is a central mechanism in PCM-induced renal and testicular toxicity, characterized by impaired antioxidant defenses and increased lipid peroxidation (Adebajo et al., 2024). Although nuclear factor erythroid 2–related factor 2 (Nrf2) and its downstream target heme oxygenase-1 (HO-1) normally regulate redox homeostasis, PCM toxicity disrupts this pathway, leading to reduced superoxide dismutase (SOD), catalase (CAT), and GSH levels, alongside elevated malondialdehyde (MDA) (El-Maddawy and El-Sayed, 2018; Elsafty et al., 2024). In parallel, oxidative stress activates nuclear factor-kappa B (NF-κB), increasing the expression of pro-inflammatory cytokines, particularly tumor necrosis factor-alpha (TNF-α), which further exacerbates tissue injury (Ansari et al., 2020). The combined effects of oxidative stress and inflammation ultimately promote apoptosis through NF-κB–dependent TNF-α signaling, favoring Bax activation and Bcl-2 downregulation (Aksu et al., 2016; Al-Rasheed et al., 2017).

In the kidney, excessive NAPQI accumulation and redox imbalance result in structural and functional injury, accompanied by the upregulation of sensitive renal injury biomarkers, including neutrophil gelatinase-associated lipocalin (NGAL) and kidney injury molecule-1 (KIM-1), which reflect oxidative stress-induced apoptosis and tubular damage (Adil et al., 2016b; Akakpo et al., 2020). Similarly, PCM-induced testicular toxicity is mediated by oxidative damage and apoptosis, leading to marked histopathological alterations, sperm abnormalities, impaired sperm parameters, and hormonal disturbances (Aksu et al., 2016; Diab et al., 2020). Previous studies have also reported reduced testosterone levels, altered follicle-stimulating hormone (FSH) and luteinizing hormone (LH), and dysregulation of key steroidogenic genes such as steroidogenic acute regulatory protein (StAR) and 3β-hydroxysteroid dehydrogenase (3β-HSD) following PCM intoxication (Olaniyi and Agunbiade, 2018; Khayyat, 2021; Blecharz-Klin et al., 2022).

N-acetyl cysteine (NAC) remains the only approved antidote for PCM toxicity, primarily through replenishment of intracellular GSH stores (Akakpo et al., 2021). Nevertheless, its clinical use is limited by adverse reactions, including nausea, hypotension, and anaphylactoid responses (Fatima et al., 2022). Moreover, delayed presentation may still result in renal failure despite NAC administration, and experimental evidence suggests variable or limited nephroprotective efficacy in some models (Alqahtani et al., 2023). Although NAC has shown some protective effects against PCM-induced testicular damage (El-Maddawy and El-Sayed, 2018), these limitations highlight the need for safer and more effective therapeutic alternatives.

In this context, natural antioxidants have gained increasing attention due to their safety profile and broad biological activities (Edo et al., 2023). *Lagerstroemia speciosa* (L.) Pers. (*L.speciosa*) has been reported to exert potent antioxidant effects (Pal et al., 2020), attributed to its rich phytochemical composition, including γ-sitosterol, phytol, and α-tocopherol (Sirikhansaeng et al., 2017), as well as triterpenoids, ellagitannins, ellagic acid, and corosolic acid (Amresh et al., 2018). Despite these documented properties, its potential protective role against PCM-induced renal and testicular toxicity has not yet been investigated.

Therefore, given that PCM-induced renal and testicular injuries are driven by oxidative stress, inflammatory responses, and apoptosis (Dallak et al., 2022), the present study is the first to evaluate the protective effects of L. speciosa in both organs simultaneously. Furthermore, it compares the efficacy of *L. speciosa* with NAC, focusing on key molecular pathways involved in redox regulation, inflammation, apoptosis, and organ injury, including Nrf2–HO-1, NF-κB–TNF-α, Bax–Bcl-2, KIM-1, and NGAL.

## 2 Materials and methods

### 2.1 Drugs

Paracetamol and N-acetyl cysteine were obtained from Sigma-Aldrich (United States) (Product Codes: 1003637432 and 102604034, respectively).

### 2.2 Plant collection

Fresh leaves of *L. speciosa* were harvested in June 2022 from a nursery located in Daqahlia Governorate, Egypt. The plant sample was authenticated taxonomically by experts at the Faculty of Agriculture, Mansoura University.

### 2.3 Preparation of ethanolic extract

Fresh *L. speciosa* leaves were collected, thoroughly rinsed with purified water to eliminate surface contaminants, and subsequently air-dried in the shade for approximately 1 month. The dried material was then pulverized, and a fine powder was prepared through the use of an automated grinder. For extraction, 50 g of the powder was subjected to continuous hot extraction in a Soxhlet apparatus using 250 mL of absolute ethanol at 60 °C for 6 h. The resulting extract was concentrated by evaporating the solvent utilizing a rotovap, followed by lyophilization at −40 °C under high vacuum to obtain a dry powdered extract (Amresh et al., 2018). The procedure yielded 14.2 g of extract per 100 g of dry leaf material, corresponding to a 14.2% extraction yield. The final ethanolic extract of *L. speciosa* leaves (EELS) was stored in airtight containers at −20 °C for later use.

### 2.4 Chemical profiling of EELS using gas chromatographic–mass spectrometric analysis

Phytochemical screening of EELS was conducted using gas chromatography combined with mass spectrometry (GC-MS) to characterize its chemical constituents. The procedure was conducted in line with the settings described by El-Kareem et al. (2016), including column specifications, temperature programming, and ionization settings.

### 2.5 Experimental animals

Male albino rats (n = 40), aged 8 weeks and weighing between 180 and 200 g, were sourced from the Laboratory Animal Facility at Zagazig University, Egypt. Experimental animals were maintained within polypropylene housing cages with controlled laboratory conditions, including regular alternating light and dark periods and regulated temperature. Free access to a standard diet and clean water was available throughout the study. The approval of all experimental protocols was granted by the Institutional Animal Ethics Board at the Faculty of Veterinary Medicine, Mansoura University, Egypt (Ethical Approval Code: M/152).

### 2.6 Animal treatment

**FIGURE 1:**
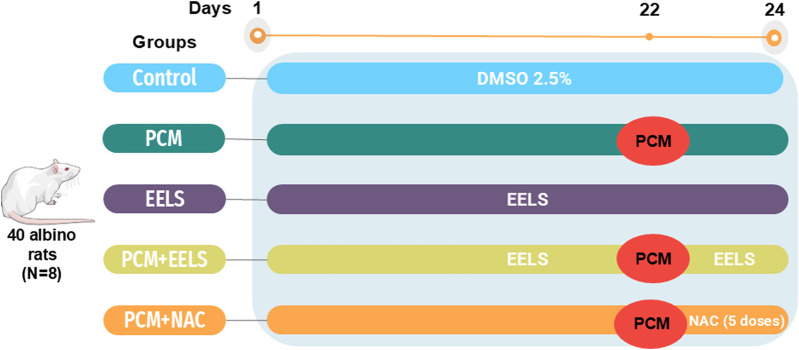
Schematic representation of the experimental design and treatment timeline of PCM-intoxicated rats treated with EELS or NAC. Rats were allocated into five groups: Control, PCM, EELS, PCM + EELS, and PCM + NAC. PCM was administered as a single oral dose on day 22, while EELS was given orally throughout the experimental period. NAC was administered intraperitoneally starting 2 hours after PCM and continued every 12 h for five doses. PCM (paracetamol); EELS (ethanolic extract of *Lagerstroemia speciosa* leaves); NAC (N-acetyl cysteine).

After a 14-day acclimatization period, Wistar albino rats were randomized into five groups of eight (the experimental design and treatment timeline are illustrated in Figure 1). The control group received oral 2.5% dimethyl sulfoxide (DMSO) (vehicle of EELS) throughout the study (24 days). The paracetamol (PCM) group was fasted overnight, and PCM was given orally as a single dose (2 g/kg b. w) on day 22 after dissolution in sterile saline and warming of the solution to 65 °C (Latif et al., 2021). The ethanolic extract of *L. speciosa* (EELS) group received 500 mg/kg b. w of EELS, a dose selected based on previous studies demonstrating its safety and antioxidant efficacy in drug-induced toxicity rat models, and was administered orally once daily in 2.5% DMSO throughout the experimental period (Rohit Singh et al., 2021). The PCM + EELS group received both PCM and EELS as described. In the PCM + NAC group, NAC was administered intraperitoneally at a dose of 100 mg/kg b. w, starting 2 hours after PCM and subsequently every 12 h for a total of five doses until the end of the experiment. The dose and dosing regimen were selected based on previously validated APAP toxicity models demonstrating biological and mechanistic relevance in rodent studies (Akakpo et al., 2021; Elshal and Abdelmageed, 2022). All oral treatments were delivered via gavage.

### 2.7 Collection of samples

Forty-eight hours after PCM administration, anesthesia was induced in the animals with ketamine (50 mg/kg b. w) combined with xylazine (10 mg/kg b. w), administered intraperitoneally (Bhatia et al., 2022). Blood samples were collected through the medial orbital canthus into plain tubes, left to clot, and then centrifuged at 1198 *g* for 10 min to obtain serum, which was stored at −20 °C until biochemical assessment. After animals were humanely euthanized by cervical dislocation, kidney and testicular tissues were excised. Portions of each organ were immediately fixed in 10% neutral-buffered formalin for histopathological investigations. Separate portions of the kidneys and testes underwent homogenization in phosphate buffer saline and centrifuged at 12,000 × *g* for 10 min; the supernatant fluids were preserved at −20 °C for the measurement of oxidative stress biomarkers, including MDA and GSH. Additionally, a small fragment of each tissue was used for RNA extraction and subsequent quantitative gene expression analysis.

### 2.8 Epididymal sperm collection and evaluation

Spermatozoa were collected immediately after euthanasia from the right epididymis. The tissue was gently minced in 1 mL of isotonic saline using fine scissors, followed by gentle maceration with forceps for approximately 2 min to facilitate sperm release. The suspension was then incubated at 25 °C for 4 h to allow sperm to migrate into the medium, as previously described by Türk et al. (2008). After incubation, the mixture was passed through a fine nylon mesh strainer (∼70 μm pore size) to remove tissue fragments, and the clarified supernatant was used for further sperm assessments, including sperm count, viability (percentage of dead sperm), and morphological evaluation.

The determination of sperm count was performed with a hemocytometer according to Yokoi et al. (2003). Briefly, the sperm suspension was diluted 1:200 with a staining solution containing eosin, formalin, and sodium bicarbonate in distilled water. Ten microliters of the diluted sample were introduced into an improved Neubauer chamber (0.1 mm depth) using a red blood cell counting pipette, left to settle for 5 min, and counted under a light microscope at 200× magnification.

Sperm morphology was assessed according to Türk et al. (2008), with a modification in staining procedure, in which aqueous eosin-Y at 0.05% concentration was used instead of the eosin–nigrosin combination. Prepared smears were subjected to staining and then examined at 400× magnification. For each slide, 300 spermatozoa were evaluated, and structural abnormalities of the head, midpiece, and tail were recorded. All sperm analyses were performed by an investigator blinded to the experimental group allocation.

### 2.9 Assessment of reproductive hormone profiles and serum renal function parameters

Serum levels of reproductive hormones, including testosterone, follicle-stimulating hormone (FSH), and luteinizing hormone (LH), were measured using rat-specific ELISA kits (Kamiya Biomedical Company, Seattle, WA, United States; catalog numbers KT-49532, KT-25338, and KT-13065, respectively).

Renal function biomarkers in serum were analyzed using a spectrophotometer (BM, Germany, 5010) with commercial assay kits, including creatinine [Ref. 10051] from Human (Wiesbaden, Germany); urea [Ref. TK41041] and phosphorus [Ref. 1101150] from Spinreact (Sant Esteve de Bas, Spain); and uric acid [Ref. 323001] from Spectrum Diagnostics (Cairo, Egypt). Concentrations of serum electrolytes, including calcium, sodium, and potassium, were determined using an AFT-500 electrolyte analyzer (Cornley, Meizhou Cornley Hi-Tech Co., Shenzhen, China).

### 2.10 Estimation of renal and testicular MDA and GSH

MDA and GSH levels in renal and testicular samples were quantified using spectrophotometric analysis with commercially available kits from Bio-Diagnostic (Cairo, Egypt) (Batch Nos. 240505 and 230501, respectively), following the manufacturer’s recommended protocols.

### 2.11 Analysis of gene expression in renal and testicular tissue samples

Renal and testicular tissues were subjected to total RNA extraction with the RNeasy Mini Kit (Qiagen, Cat. No. 74104) in line with the manufacturer’s recommendations. RNA purity and concentration were checked spectrophotometrically, and complementary DNA (cDNA) was synthesized with RevertAid Reverse Transcriptase (Thermo Fisher). Quantitative real-time polymerase chain reaction (qRT-PCR) was carried out on a Stratagene MX3005P system using the QuantiTect SYBR Green PCR Kit (Qiagen, Cat. No. 204141). Gene expression was assessed for steroidogenic regulators (3β-HSD and StAR) in testicular tissues, as well as renal injury biomarkers (KIM-1 and NGAL) in kidney samples. In addition, antioxidant-related genes (Nrf2, HO-1, SOD, and CAT), inflammation-associated markers (NF-κB and TNF-α), and apoptosis-related targets (Bax and Bcl-2) were evaluated in renal and testicular tissues. β-actin served as the internal control gene. Amplification was conducted under kit-recommended cycling conditions, and the 2^−ΔΔCT^ method was employed to determine gene expression fold changes (Pfaffl, 2001).

### 2.12 Histological and histomorphometric examination of renal and testicular tissues

Tissue specimens were fixed in 10% neutral buffered formalin for 24 h, dehydrated in graded ethanol, cleared in xylene, and embedded in paraffin. Sections of 5 µm were prepared with a rotary microtome, mounted on charged slides, and stained with hematoxylin and eosin (H&E) following standard protocols (Bancroft and Floyd, 2012). Briefly, paraffin was removed with xylene, tissues were rehydrated through descending alcohol concentrations, nuclei were stained with hematoxylin, counterstained with eosin, dehydrated, cleared, and mounted in DPX for histological evaluation. Quantitative histological assessments were carried out using ImageJ software (version 1.36; NIH, United States). From each experimental group, three animals were randomly selected, and for every animal, five stained tissue sections were analyzed at 40× magnification, ensuring non-overlapping fields. In kidney samples, measurements included mean glomerular diameter, glomerular cellularity, and the percentage of degenerated renal tubules (Hamdy et al., 2024). Testicular morphometry involved determining the epithelial thickness of seminiferous tubules and the density of seminiferous epithelial cells (Samy et al., 2020). The progression of germ-cell development within seminiferous tubules was further assessed using the Johnsen scoring system, which ranks spermatogenesis on a scale of 1 (absence of seminiferous epithelium) to 10 (complete spermatogenic activity) (Johnsen, 1970; Ozmerdiven et al., 2017).

### 2.13 Statistical analysis

The dataset was processed using SPSS software (IBM, version 20.0, United States). Prior to parametric analysis, normality and homogeneity of variance were assessed using the Kolmogorov–Smirnov and Levene’s tests, respectively. Differences among groups were then examined through one-way analysis of variance (ANOVA). Post-hoc comparisons were primarily performed using the Least Significant Difference (LSD) test following a significant ANOVA. Duncan’s multiple range test was additionally used for grouping means into homogeneous subsets and assigning significance letters for presentation purposes only. A probability value of *p* < 0.05 was considered statistically significant. Results are expressed as mean ± standard deviation (SD) for all datasets except histopathological and sperm analysis data, which are presented as mean ± standard error of the mean (SEM), as specified in the figure legends and table footnotes. Graphs were generated using Microsoft Excel 2016.

## 3 Results

### 3.1 Major constituents of EELS identified via GC-MS

The analysis of EELS identified several major chemical constituents. These included fatty acids (n-hexadecenoic acid, oleic acid, and 9,12,15-octadecatrienoic acid), sterols (campesterol, stigmasterol, and ç-sitosterol), triterpenes (e.g., squalene, phytol and α-tocopherol). Compound identification was based on spectral comparison with the NIST 14 and WILEY 09 mass spectral databases.

### 3.2 Evaluation of epididymal spermatozoa

**FIGURE 2:**
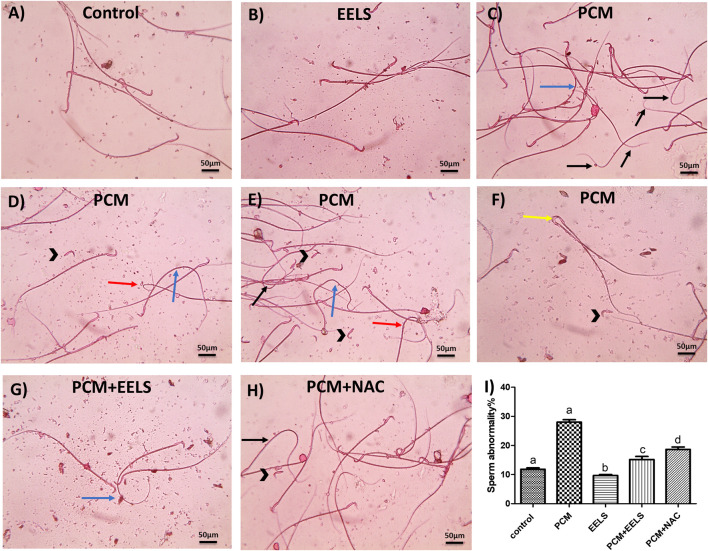
Semen smear stained with 0.05% aqueous solution of eosin-Y illustrating morphologically normal sperm, including head, body, and tail, under the light microscope at ×400 magnification in the control normal group **(A)** and the EELS group **(B)**. Several morphological abnormalities are seen in the prepared smears from the PCM group **(C–F)**, including detached head (black arrowhead), curved tail (blue arrow), bent tail (black arrow), looped tail (red arrows), and fused heads (yellow arrows). Smears from the PCM + EELS group **(G)** showing very few sperm morphological abnormalities, including curved tail (blue arrow). Also, smears from the PCM + NAC group **(H)** show few sperm morphological abnormalities, including detached heads (black arrowheads) and bent tails (black arrow). **(I)** Bars are mean ± SEM showing total sperm abnormality %. Different superscript letters indicate significant differences (*p* < 0.05). PCM (paracetamol); EELS (*Lagerstroemia speciosa* extract); NAC (N-acetyl cysteine).

Sperm counts were not significantly altered across the groups; however, the proportion of non-viable sperm was markedly higher in PCM-treated rats, an effect that was clearly reduced by co-treatment with either EELS or NAC (Table 1). Microscopic evaluation of stained semen smears reinforced these results. In the control (Figure 2A) and EELS-only groups (Figure 2B), spermatozoa largely retained normal morphology, showing distinct heads, midpieces, and tails. By contrast, samples from PCM-treated rats (Figures 2C–F) exhibited a sharp increase in structural abnormalities, including fused or detached heads and tails that appeared bent, looped, or irregularly curved. These defects were much less evident in the PCM + EELS group (Figure 2G), where only occasional mild tail deformities were noted. Likewise, the PCM + NAC group (Figure 2H) showed an apparent reduction in abnormalities, primarily limited to detached heads and bent tails. Statistical comparisons confirmed these morphological findings, revealing a significant rise (p < 0.05) in abnormal sperm percentages across groups (Figure 2I; Table 2).

**TABLE 1:** Effect of *Lagerstroemia speciosa* extract on PCM-induced changes in sperm count, viability, and morphological abnormalities.

Parameters	Experimental groups				
	Control	PCM	EELS	PCM + EELS	PCM + NAC
Sperm count (x10^6^/ml)	102.33 ± 11.68^a^	96.67 ± 11.59^a^	105.00 ± 7.55^a^	99.00 ± 3.61^a^	100.33 ± 7.09^a^
Dead sperm rate (%)	10.00 ± 1.00^c^	25.33 ± 3.06^a^	10.33 ± 1.53^c^	16.67 ± 1.53^b^	15.67 ± 1.53^b^
Abnormal sperm head *(%)*	1.00 ± 0.3^b^	4.8 ± 0.4^a^	1.1 ± 0.1^b^	1.3 ± 0.2^b^	1.5 ± 0.2^b^
Abnormal sperm mid *(%)*	0.3 ± 0.2^b^	3.33 ± 0.3^a^	0.5 ± 0.2^b^	0.6 ± 0.2^b^	0.8 ± 0.3^b^
Abnormal sperm tail *(%)*	0.4 ± 0.2^c^	12.33 ± 0.8^a^	0.7 ± 0.2^c^	4.3 ± 0.6^b^	4.6 ± 0.3^b^
Total sperm abnormality *(%)*	1.5 ± 0.5^c^	20.5 ± 1.11^a^	2.16 ± 0.4^c^	6.3 ± 1.06^b^	7.00 ± 0.5^b^

Data are presented as mean ± SEM (n = 8). Within each row, values annotated with different superscript letters differ significantly (*p* < 0.05). PCM (paracetamol); EELS (*L. speciosa* extract); NAC (N-acetyl cysteine).

**TABLE 2:** Modulation of serum renal markers and reproductive hormone profiles by *Lagerstroemia speciosa* extract in PCM-intoxicated rats.

Parameters	Experimental groups				
	Control	PCM	EELS	PCM + EELS	PCM + NAC
Testesterone *(ng/mL)*	5.28 ± 0.89^a^	1.39 ± 0.43^c^	6.02 ± 1.21^a^	2.58 ± 0.42^b^	3.02 ± 0.11^b^
FSH *(ng/mL)*	3.27 ± 0.26^a^	3.38 ± 0.39^a^	3.30 ± 0.44^a^	3.20 ± 0.36^a^	3.37 ± 0.43^a^
LH *(ng/mL)*	1.72 ± 0.08^c^	2.48 ± 0.15^a^	1.66 ± 0.10^c^	1.83 ± 0.27^bc^	2.06 ± 0.22^b^
Creatinine *(mg/dL)*	0.55 ± 0.05^c^	0.89 ± 0.08^a^	0.51 ± 0.07^c^	0.69 ± 0.05^b^	0.68 ± 0.03^b^
Urea *(mg/dL)*	30.27 ± 1.05^c^	47.78 ± 2.59^a^	31.45 ± 0.61^c^	38.98 ± 2.65^b^	35.87 ± 3.09^b^
Uric acid *(mg/dL)*	1.56 ± 0.13^c^	3.66 ± 0.50^a^	1.50 ± 0.25^c^	2.40 ± 0.34^b^	2.58 ± 0.35^b^
Phosphorus *(mg/dL)*	4.66 ± 0.16^c^	7.21 ± 0.38^a^	4.61 ± 0.33^c^	5.46 ± 0.68^b^	5.58 ± 0.40^b^
Calcium *(mg/dL)*	11.47 ± 0.38^a^	8.94 ± 0.47^c^	11.27 ± 0.52^a^	10.39 ± 0.56^b^	10.80 ± 0.38^ab^
Sodium *(mmol/L)*	134.88 ± 0.63^c^	145.70 ± 3.00^a^	135.75 ± 0.56^c^	140.13 ± 2.17^b^	139.68 ± 4.24^b^
Potassium *(mmol/L)*	5.78 ± 0.61^a^	3.83 ± 0.41^c^	5.76 ± 0.52^a^	4.28 ± 0.17^bc^	4.63 ± 0.46^b^

Data are presented as mean ± SD (n = 8). Within each row, values annotated with different superscript letters differ significantly (*p* < 0.05). FSH (follicle-stimulating hormone); LH (leutenizing hormone); PCM (paracetamol); EELS (*L. speciosa* extract); NAC (N-acetyl cysteine).

### 3.3 Serum reproductive hormone profiles and renal function parameters

As presented in Table 2, PCM administration resulted in a sharp reduction in serum testosterone, while LH levels were significantly elevated; however, FSH levels did not differ among groups. Co-treatment with either EELS or NAC substantially improved these hormonal alterations, bringing them closer to control values, with no statistically significant differences between the two treatments. Statistical significance for intergroup comparisons was set at p < 0.05.

Serum creatinine, urea, and uric acid levels were comparable between the control and EELS groups; however, they showed a significant elevation in PCM-treated rats, indicating renal dysfunction. PCM exposure also produced marked increases in serum phosphorus and sodium, accompanied by declines in Calcium and potassium. Co-treatment with either EELS or NAC effectively counteracted these disturbances, restoring the values toward normal.

### 3.4 Renal and testicular MDA and GSH

**FIGURE 3:**
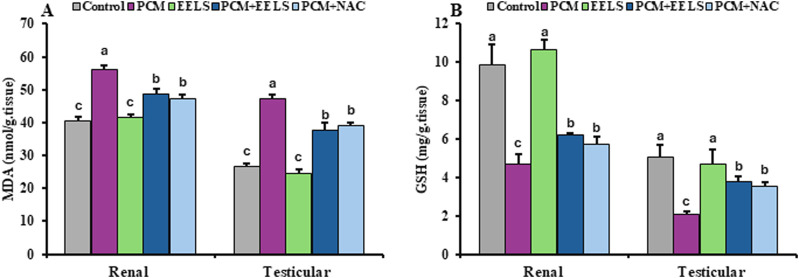
**(A)** MDA and **(B)** GSH levels in renal and testicular tissues of PCM-intoxicated rats treated with EELS or NAC. Data are presented as mean ± SD (n = 8). Data were analyzed by one-way ANOVA with Duncan’s multiple range test (*p < 0.05*). Columns not sharing the same superscript letter differ significantly. Superscripts are also shown above the control group if its mean value significantly differs from other groups. MDA (malondialdehyde); GSH (reduced glutathione); PCM (paracetamol); EELS (*Lagerstroemia speciosa* extract); NAC (N-acetyl cysteine).

PCM exposure resulted in a pronounced increase in MDA, accompanied by a reduction in GSH in both renal and testicular tissues. Co-administration of EELS or NAC substantially alleviated these oxidative changes by reducing MDA accumulation and increasing GSH levels; however, no statistically significant differences were observed between the two treatments, and the values did not completely return to those of the control group (Figure 3).

### 3.5 Gene expression in renal and testicular tissue samples

**FIGURE 4:**
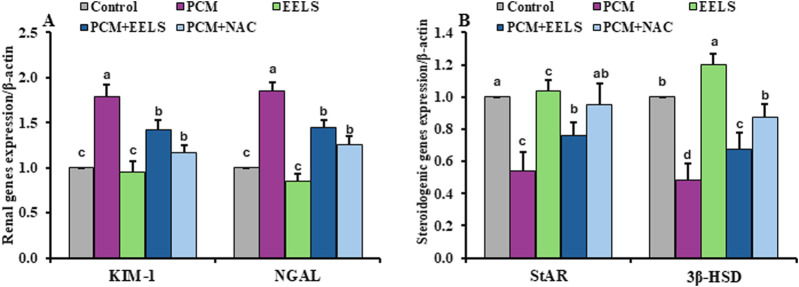
Levels of mRNA expression of renal injury markers and steroidogenic pathway genes in renal and testicular tissues of PCM-intoxicated rats treated with EELS or NAC. Data are presented as mean ± SD (n = 8). Data were analyzed by one-way ANOVA with Duncan’s test (*p < 0.05*). Bars not sharing a superscript differ significantly, including the control when its mean differs from other groups. **(A)** KIM-1 (kidney injury molecule-1); NGAL (neutrophil gelatinase-associated lipocalin). **(B)** StAR (steroidogenic acute regulatory protein); 3β-HSD (3β-hydroxysteroid dehydrogenase). PCM (paracetamol); EELS (*Lagerstroemia speciosa* extract); NAC (N-acetyl cysteine).

**FIGURE 5:**
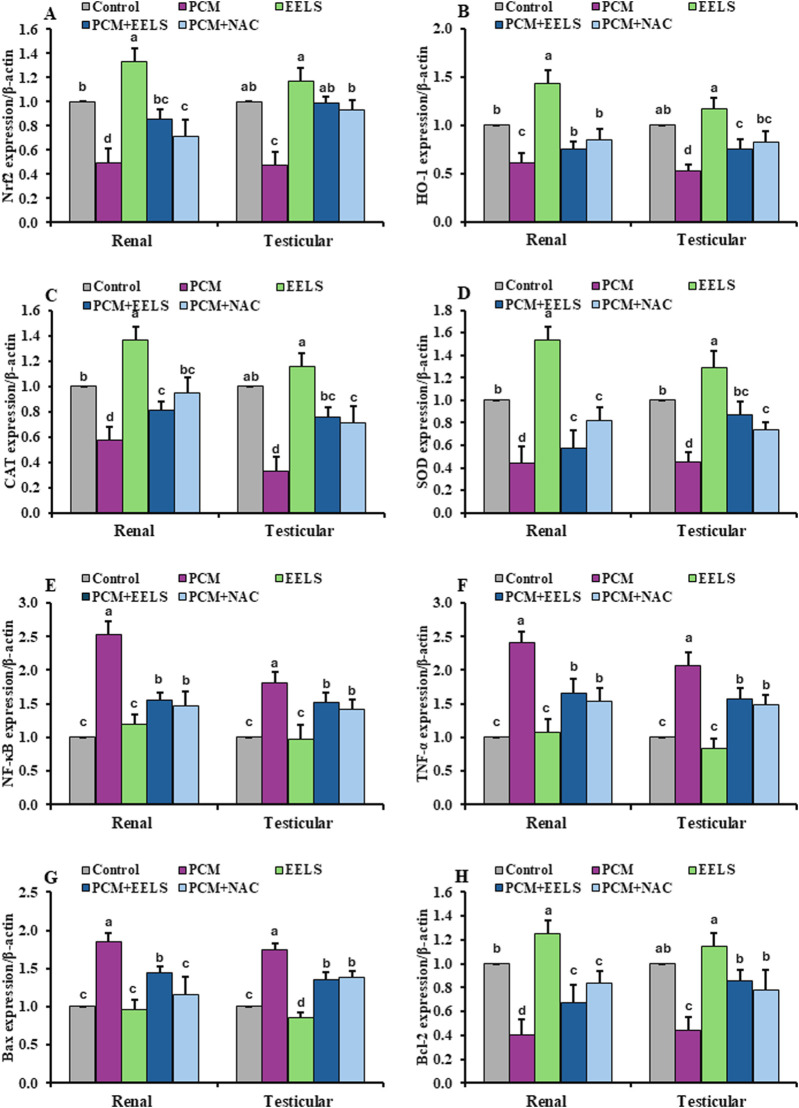
Levels of mRNA expression of antioxidant, pro-inflammatory, apoptotic, and anti-apoptotic markers in renal and testicular tissues of PCM-intoxicated rats treated with EELS or NAC. Data are presented as mean ± SD (n = 8). Data were analyzed by one-way ANOVA with Duncan’s multiple range test (*p < 0.05*). Columns not sharing the same superscript letter differ significantly. Superscripts are also shown above the control group if its mean value significantly differs from other groups. **(A)** Nrf2 (nuclear factor erythroid 2-related factor 2); **(B)** HO-1 (heme oxygenase-1) **(C)** CAT (catalase); **(D)** SOD (superoxide dismutase); **(E)** NF-κB (nuclear factor kappa B); **(F)** TNF-α (tumor necrosis factor-alpha); **(G)** Bax (Bcl-2–associated X protein); **(H)** Bcl-2 (B-cell lymphoma-2). PCM (paracetamol); EELS (*Lagerstroemia speciosa* extract); NAC (N-acetyl cysteine).

As illustrated in Figure 4, PCM intoxication markedly downregulated the transcriptional levels of steroidogenesis-related genes StAR and 3β-HSD, while upregulating renal injury–associated genes KIM-1 and NGAL. As shown in Figure 5, PCM overdose suppressed antioxidant-related genes, including the components of the Nrf2/HO-1 pathway, CAT and SOD in both kidney and testis samples, and induced the inflammatory genes NF-κB and TNF-α. Furthermore, rats in the PCM group had elevations in the pro-apoptotic marker Bax and diminished Bcl-2 expression. Additionally, these alterations were noticeably improved in the PCM + EELS and PCM + NAC groups, with gene expression levels tending to return closer to those seen in the control group. No statistically significant differences were detected between EELS and NAC treatments in PCM-intoxicated rats for most genes; however, NAC produced a significantly greater downregulation of the pro-apoptotic gene Bax.

### 3.6 Histological and histomorphometric findings in renal and testicular tissues

**FIGURE 6:**
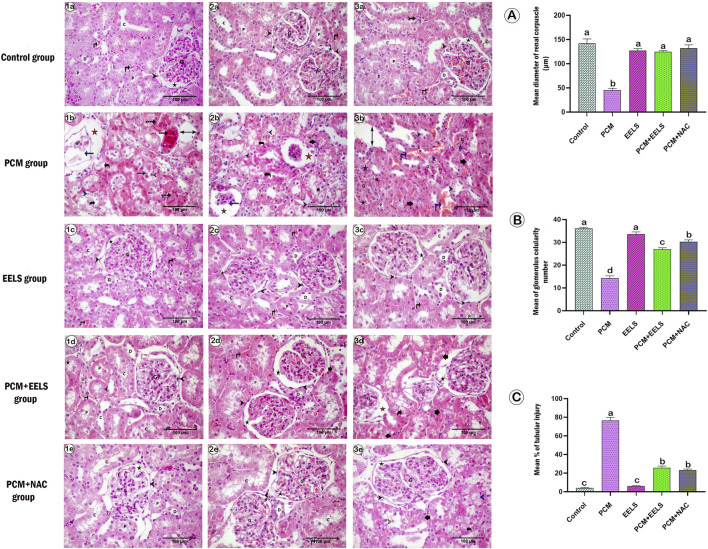
Representative photomicrograph of H&E-stained renal sections in different groups: (a1-3) control group, (b1-3) PCM group, (c1-3) EELS group, (d1-3) PCM + EELS group, and (e1-3) PCM + NAC group. PCM (paracetamol); EELS (*Lagerstroemia speciosa* extract); NAC (N-acetyl cysteine). Normal capsular space of renal corpuscle (black asterisk), tuft of glomerulus capillary (G), proximal (P) and distal (d) convoluted tubules and collecting (c) tubule with light vesicular circular nuclei, small area of interstitial cells (tailed arrow), normal appearance blood vessels (black vertical arrow), Distorted and shrunken renal corpuscles with few or completely atrophied glomerulus capillaries (discontinues blue arrow) and wide capsular space (orange asterisk), degenerated renal corpuscle with complete closure capsular space (blue asterisk) Edematous and dilated blood vessels (blue vertical arrow), degenerated tubules(short black arrow), degenerated tubules with vacuolated epithelial lining (curved black arrow), degenerated tubules with desquamated epithelial cells (green arrow head) or chromatolytic nuclei (blue arrow head). **(A–C)** represent the mean diameter of renal corpuscle (micron), mean number of glomeruli cells, and mean percent of renal tubular injury, respectively. Data are expressed as mean ± SEM. The different letters (a, b, and **(C)** indicate marked differences between experimental groups.

Control rats (Figure 6, 1a–3a) and those given EELS alone (Figure 6, 1c–3c) displayed preserved cortical architecture with intact glomeruli and tubules. In contrast, PCM-treated animals (Figure 6, 1b–3b) exhibited marked pathological lesions, including dilated Bowman’s spaces, partial or complete glomerular atrophy, and vacuolated tubular epithelium. Quantitative evaluation confirmed these findings, showing a significant reduction in glomerular diameter and cellularity (Figures 6A,B) together with a sharp rise in tubular injury percentage (Figure 6C). Co-administration of EELS (Figure 6,1d–3d) or NAC (Figure 6,1e–3e) notably mitigated these lesions, with corpuscular dimensions, cellularity, and tubular scores largely restored toward control levels.

**FIGURE 7:**
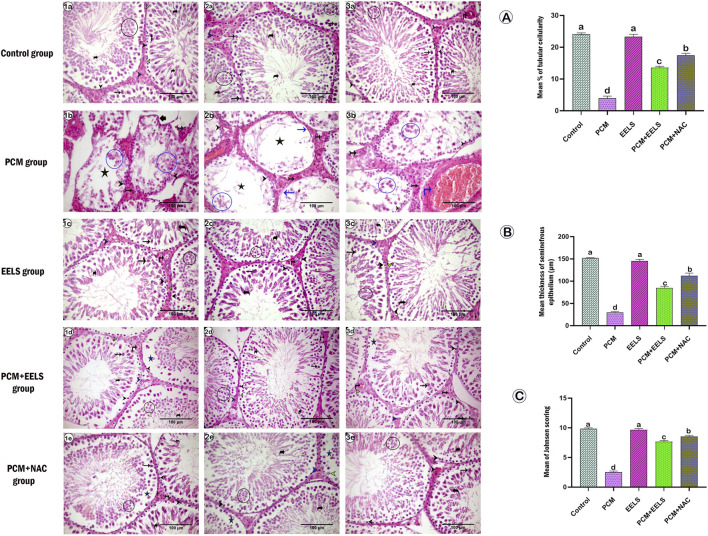
Representative photomicrograph of H&E-stained testicular sections in different groups: (a1-3) control group, (b1-3) PCM group, (c1-3) EELS group, (d1-3) PCM + EELS group, and (e1-3) PCM + NAC group. PCM (paracetamol); EELS (*Lagerstroemia speciosa* extract); NAC (N-acetyl cysteine). Seminiferous tubules lined with spermatogonia (black arrow head), Sertoli cells (green arrow head), primary spermatocytes (black arrow), early spermatid (inside black circle), spermatozoa (curved arrow), interstitial Leydig cells (blue arrow head), normal blood vessels (vertical black arrow), seminiferous tubules with irregular basement membrane (short thick arrow), seminiferous tubules with empty lumen (black asterisk), seminiferous tubule with empty area of lining epithelium (blue asterisk), basement membrane without spermatogonia or Sertoli cells (blue arrow), degenerated spermatogenic cells (inside blue circle), Leydig cells with dark pyknotic nuclei (tailed black arrow) and congested blood vessels (blue vertical arrow). **(A)** represents the mean % of epithelial cell density within each tubule. **(B)** represents the mean of epithelial thickness of each tubule. **(C)** represents the mean of Johnsen’s scoring within each tubule. Data are expressed as mean ± SEM. The different letters **(A–C)** indicate a significant difference between experimental groups.

Testicular sections from the control (Figure 7,1a–3a) and EELS groups (Figure 7,1c–3c) showed intact seminiferous tubules with normal spermatogenic layers and well-preserved interstitial tissue. In contrast, PCM-treated rats (Figure 7,1b–3b) exhibited severe disruption, with depleted germinal epithelium, pyknotic interstitial nuclei, and impaired spermatogenesis. Quantitative analysis confirmed significant reductions in epithelial cell density (Figure 7A) and thickness (Figure 7B), alongside a sharp decline in Johnsen scores (Figure 7C). Co-treatment with EELS (Figure 7,1d–3d) or NAC (Figure 7,1e–3e) restored much of the seminiferous architecture, with notable improvements in morphometric parameters, more pronounced in the NAC group.

## 4 Discussion

This research was targeted to examine the preventive actions of *L. speciosa* against PCM-induced renal and testicular damage, emphasizing oxidative damage, inflammatory response, and apoptosis. The findings indicated that PCM overdose impaired many functional and structural measures, but *L. speciosa* therapy significantly mitigated these changes, presumably via modulating the Nrf2–HO–1 axis, NF–κB–TNF–α pathway, and Bax–Bcl–2 balance, with effects that were comparable to NAC treatment.

In this work, PCM administration profoundly impaired male reproductive function, as evidenced by reduced sperm viability and abnormal morphology, decreased testosterone levels with concomitant elevation of LH, a significant reduction in Johnsen score, and suppressed expression of the steroidogenic regulators StAR and 3β-HSD. These findings are consistent with the reported anti-steroidogenic effects of PCM and its disruptive impact on semen quality and testicular function in experimental models (Khayyat, 2021; Adebajo et al., 2024). The downregulation of steroidogenic gene transcripts in the PCM group provides molecular support for the observed decline in testosterone production, in line with previous evidence that PCM exposure interferes with key regulators of androgen biosynthesis (Liu et al., 2023). Notably, despite no significant changes in total sperm count or FSH levels, sperm viability and morphology were markedly altered. This apparent discrepancy can be explained by early degenerative changes in Sertoli cells and spermatogonia, and by the approximately 50-day spermatogenic cycle in rats, suggesting that numerical changes in sperm count may not yet be detectable at this stage of exposure (Zhang et al., 2023).

EELS treatment mitigated these disturbances, resulting in enhanced sperm morphology and viability, higher Johnsen’s scores, and restoration of androgen levels, along with normalization of StAR and 3β-HSD expression. Comparable improvements in testicular function, semen characteristics, and testosterone synthesis have been reported in fluoride-exposed diabetic mice receiving *L. speciosa* leaf extract. These effects may be attributed to corosolic acid, which is a major triterpenoid constituent known for its antioxidant capacity and ability to sustain Leydig cell activity (Sm and Mahaboob Basha, 2017).

In this study, rats intoxicated with PCM exhibited pronounced elevations in serum indicators of renal dysfunction. The nephrotoxic outcome is likely linked to acute kidney injury triggered by the excessive accumulation of NAPQI, which depletes intracellular glutathione and promotes oxidative stress within renal tissue. These redox disturbances compromise glomerular filtration capacity, ultimately causing retention of nitrogenous waste products like urea and creatinine (Yashiki et al., 2004; Dua et al., 2022). Histological evaluation of the kidneys further substantiated these biochemical findings, revealing both tubular and glomerular lesions, in agreement with prior reports on PCM-induced renal damage (Canayakin et al., 2016).

Electrolyte derangements also emerged as a key feature in PCM-treated rats. The profile included hypernatremia, hypokalemia, hypocalcemia, and hyperphosphatemia, reflecting impaired tubular handling of solutes. While our findings partially overlap with those of Al-Asmari et al. (2020), who described elevated sodium and potassium after high-dose PCM exposure, several mechanisms may underlie the discrepancies. Elevated serum sodium may arise from reduced tubular responsiveness to antidiuretic hormone (ADH), leading to reduced water reabsorption and sodium retention (Yashiki et al., 2004). Conversely, low serum potassium is plausibly linked to renal loss secondary to dose-dependent tubular injury, consistent with clinical observations in PCM overdose (Pakravan et al., 2007). The reduction in calcium, together with phosphorus elevation, likely reflects disturbed calcium–phosphate homeostasis and calcium precipitation within renal tubules, a mechanism also supported by previous experimental work (El-Boshy et al., 2019).

At the mRNA expression level, markers of renal injury were also altered. KIM-1, a membrane glycoprotein involved in the elimination of apoptotic debris (Adil et al., 2016b), and NGAL, a cytosolic protein released from damaged renal tubules (Adil et al., 2016a), were both significantly upregulated following PCM insult. These findings are consistent with earlier studies highlighting KIM-1 and NGAL as sensitive early biomarkers of renal parenchymal damage (Ozatik et al., 2019; Hamdy et al., 2024).

Significantly, treatment with EELS substantially counteracted these PCM-induced alterations, restoring renal function parameters, correcting electrolyte imbalances, and downregulating injury biomarkers. This protective role echoes prior reports of *L. speciosa* safeguarding against diabetic nephropathy (Mahaboob Basha and Saumya, 2013) and cisplatin-induced kidney injury (Priya et al., 2007). The nephroprotective efficacy of the extract is likely mediated by its flavonoid-rich phytochemical profile, which is known for its potent free radical scavenging activity and the attenuation of reactive oxygen species (ROS)-driven tissue damage (Aljarba et al., 2021).

Oxidative stress and ROS generated by NAPQI are widely recognized as key contributors to PCM-induced tissue damage (Elsafty et al., 2024). PCM administration in this study has resulted in pronounced oxidative stress in the kidney and testis, as demonstrated by GSH depletion and increased MDA levels, indicating oxidative imbalance in both organs by the PCM toxic metabolite, NAPQI (El-Maddawy and El-Sayed, 2018). This redox imbalance was additionally validated through the observed downregulation of key antioxidant defence genes in our study, including Nrf2, which regulates a broad spectrum of antioxidant genes, including HO-1, CAT, and SOD, suggesting suppression of the cellular oxidative stress response system due to PCM overdose (Canayakin et al., 2016; Alqahtani et al., 2023). Our results regarding PCM-induced oxidative stress align with earlier reports (Adil et al., 2016b; Elsafty et al., 2024; Diab et al., 2025; Shi et al., 2025).

In this study, EELS significantly reduced MDA and restored GSH in both organs of PCM-intoxicated rats, reflecting strong antioxidant activity. It also upregulated the expression of Nrf2, HO-1, SOD, and CAT. The consistent protection in renal and reproductive tissues suggests potent antioxidant effects of *L. speciosa* (Rohit Singh et al., 2021). This may be attributed to its natural antioxidant properties, which function either through the direct neutralization of ROS and/or by enhancing the endogenous cellular antioxidant defence system via upregulation of Nrf2 (Sahu et al., 2015). Our findings are consistent with earlier reports on *L. speciosa*’s role in alleviating oxidative damage in diabetic nephropathy in rats (Aljarba et al., 2021) and cardiotoxicity in mice (Sahu et al., 2015).

Inflammatory responses in PCM intoxication are considered secondary to oxidative stress, as surplus ROS initiates mediator release that further aggravates tissue and organ pathology (Alqahtani et al., 2023). A key regulator of this process, the transcription factor NF-κB, is activated under oxidative stress, relocates to the nucleus, and facilitates the induction of inflammatory cytokine synthesis, including TNF-α (El-Kashef and Sharawy, 2023). In this study, PCM administration led to a remarkable increase in NF-κB and TNF-α genes in both renal and testicular tissues, reflecting a pronounced inflammatory response. Histological examination supported these findings, revealing hallmark inflammatory alterations such as vascular congestion, epithelial shedding, nuclear degeneration, and pyknotic interstitial cells. These results corroborate earlier reports demonstrating NF-κB/TNF-α pathway activation in PCM-induced toxicity (Eraky and Abo El-Magd, 2020). Remarkably, treatment with *L. speciosa* leaf extract in PCM-exposed animals substantially mitigated these inflammatory responses, as evidenced by the downregulation of NF-κB and TNF-α and the near-normalization of renal and testicular tissue architecture. The anti-inflammatory efficacy of *L. speciosa* is likely mediated by its bioactive constituents such as corosolic acid, gallic acid, and berberine, which are reported to modulate inflammation-driven signaling cascades, including the NF-κB/TNF-α axis (Rohit Singh et al., 2021). Consistent with prior studies, *L. speciosa* leaf extract has been shown to restore kidney structure and suppress inflammatory markers in diabetic nephropathy models (Aljarba et al., 2021) and to alleviate inflammatory histopathological alterations in the testicular tissue of fluoride-exposed diabetic mice (Sm and Mahaboob Basha, 2017).

Bcl-2, an anti-apoptotic protein, promotes cell survival, while Bax, a pro-apoptotic protein from the same family, triggers apoptosis (Al-Rasheed et al., 2017). In this study, PCM administration increased Bax and decreased Bcl-2 expression in renal and testicular tissues. Histopathological data in our study supported this, showing glomerular atrophy, tubular degeneration, nuclear fragmentation, and vacuolation in kidneys, and thinning of seminiferous epithelium, reduced cellularity, and pyknotic interstitial nuclei in testes, indicating apoptotic damage. Previous studies showed that PCM overdose disrupts mitochondrial membrane potential, activates caspase-3, and triggers apoptosis (Elsafty et al., 2024). In our research, the Bax/Bcl-2 imbalance observed in renal tissues is in line with the findings of Al-Rasheed et al. (2017). In contrast, the testicular alterations correspond with those reported by Aksu et al. (2016). Notably, we noticed that EELS administration mitigated PCM-induced histopathological damage, lowered Bax, and upregulated Bcl-2 mRNA expression levels. Its anti-apoptotic effect likely stems from its antioxidant properties, which reduce ROS and protect mitochondrial function, aligning with Sahu et al. (2015), who reported similar changes of *L. speciosa* extract on Bax and Bcl-2 in cardiotoxic mice.

While NAC effectively served as an antidote to PCM toxicity in our study, as it demonstrated efficacy across all measured parameters, *L. speciosa* showed comparable protection. While NAC serves primarily to replenish hepatic glutathione reserves, it does not entirely prevent the nephrotoxic effects of PCM, as reactive intermediates can still provoke renal injury, thereby restricting its protective capacity in specific experimental settings (Akakpo et al., 2020). This limitation strengthens the rationale for exploring natural alternatives such as *L. speciosa* leaf extract, which appears to afford broader cytoprotection by not only enhancing antioxidant defenses but also modulating multiple molecular pathways implicated in PCM-induced extrahepatic damage.

Notably, *L. speciosa* has demonstrated a favorable preclinical safety profile, as a single oral administration of its ethanolic leaf extract at 500, 1000, 2000, and 3000 mg/kg body weight produced no signs of toxicity in rats, with preserved hepatic and renal function (Azad et al., 2015). However, the present study is preclinical in nature, and therefore direct dose translation to humans should be interpreted with caution. Although EELS was administered daily for 24 days, the use of a single dose level represents a limitation, and future dose–response studies are warranted to further define its therapeutic range. While gene expression analyses provided mechanistic insight, the lack of protein-level validation remains a limitation of the current work. In addition, the absence of pharmacokinetic data restricts conclusions regarding bioavailability and systemic exposure.

This study provides the first evidence that EELS protects renal and testicular tissues against PCM-induced injury by modulating oxidative stress, inflammation, and apoptosis. Its effects were similar to those of NAC in the present experimental model. These findings highlight the potential of *L. speciosa* as a promising preclinical candidate for mitigating PCM-associated extrahepatic toxicity. However, further studies are required to elucidate additional molecular mechanisms and to establish EELS’s translational relevance.

## Data Availability

The original contributions presented in the study are included in the article/Supplementary Material, further inquiries can be directed to the corresponding author.
