# Supplementary material for: Protective effects of Lagerstroemia speciosa against paracetamol-induced renal and testicular toxicity in rats via antioxidant, anti-inflammatory, and anti-apoptotic mechanisms
Source: Front Toxicol. 2026 Mar 4;8:1751678. doi: 10.3389/ftox.2026.1751678 (PMC12995186; doi:10.3389/ftox.2026.1751678)
Supplement: Supplementary file 1 [file Table1.docx]

**Table-1- The primer sequences utilized in the qRT-PCR examination.**

| Gene | GenBank accession number | Oligonucleotide sequence | Annealing temperature (C^0^) | Size (bp) |
| --- | --- | --- | --- | --- |
| *NF-*κ*B* | [AF079314.2](https://www.ncbi.nlm.nih.gov/entrez/viewer.fcgi?db=nucleotide&id=1125143075) | f5,- TGGACGATCTGTTTCCCCTC -3,  r5,- CCCTCGCACTTGTAACGGAA-3, | 58 | 118 |
| *TNF-α* | [NM_012675.3](https://www.ncbi.nlm.nih.gov/entrez/viewer.fcgi?db=nucleotide&id=260166688) | f5,- CTGTGCCTCAGCCTCTTCTC -3,  r5,- ACTGATGAGAGGGAGCCCAT -3, | 60 | 126 |
| *Nrf2* | NM_031144.3 | f5, - GTCCACCCGCGAGTACAACCT-3,  r5, - GGAGCCGTTGTCGACGACGA-3, | 60 | 119 |
| *HO-1* | NM_012580.2 | f5,- GCCTGGTTCAAGATACTACCTCT-3,  r5, - CTGAGTGTGAGGACCCATCG-3, | 58 | 103 |
| *SOD* | X05634.1 | f5,- GAAGGCCGTGTGCGTGCTGA -3,  f5^,^- CCTTCAGTTAATCCTGTAATC-3^,^ | 58 | 117 |
| CAT | NM_012520.2 | f5,- GGAGAGGCAGTGTACTGCAA-3,  r5,- TTGCCACTGGCGATGGCATT-3, | 58 | 140 |
| *Bax* | U32098.1 | f5,- AGGACGCATCCACCAAGAAG-3,  r5, - CAGTTGAAGTTGCCGTCTGC-3, | 58 | 166 |
| *Bcl-2* | NM_016993.2 | f5, - GGATCCAGGATAACGGAGGC-3,  r5, - ATGCACCCAGAGTGATGCAG-3, | 58 | 141 |
| *KIM-1* | AF035963.1 | f5,- AGACAGAGTGTGCTGAGTGC-3,  r5, -ACAGAGCCTGGAAGAAGCAG-3 | 59 | 121 |
| *NGAL* | NM_130741.1 | f5,- CAAGTGGCCGACACTGACTA-3,  r5, -TTCAGTTCATCGGACAGCCC-3 | 59 | 122 |
| *StAR* | NM_031558 | f5‘- GGGCATACTCAACAACCAG-3’  r5‘- ACCTCCAGTCGGAACACC-3’ | 58 | 111 |
| *3B-HSD* | M38178 | f5‘- TGTGCCAGCCTTCATCTAC-3’  r5‘- CTTCTCGGCCATCCTTTT-3’ | 56 | 145 |
| β-Actin | NM_031144.3 | f5^,^- GGCATGTGCAAGGCCGGCTT -3,  r5^,^- TAGGAGTCCTTCTGACCCATA -3^,^ | 58 | 116 |

**Table-2** Major chemical constituents identified in *Lagerstroemia speciosa* extract by GC–MS analysis.

| Retention time | Peak area% | Chemical formula | Compound class | Compound name |
| --- | --- | --- | --- | --- |
| 26.47 | 15.65% | C_16_H_32_O_2_ | Saturated fatty acid | n-Hexadecanoic acid |
| 29.52 | 14.89% | C_18_H_30_O_2_ | Polyunsaturated fatty acid (omega-3) | 9,12,15-octadecatrienoic acid |
| 29.62 | 3.85% | C_18_H_34_O_2_ | Monounsaturated fatty acid (omega-9) | Oleic acid |
| 29.19 | 3.36% | C_20_H_40_O | Terpenoid | Phytol |
| 40.19 | 9.66% | C_30_H_50_ | Terpenoid | Squalene |
| 42.99 | 6.35% | C_29_H_50_O_2_ | Terpenoid | Vitamin E (α-tocopherol) |
| 43.93 | 3.12% | C_28_H_48_O | Phytosterol | Campesterol |
| 44.23 | 1.83% | C_29_H_48_O | Phytosterol | Stigmasterol |
| 44.76 | 29.3% | C_29_H_50_O | Phytosterol | ç-Sitosterol |

Identification of compounds was based on comparison of mass spectral data with the NIST 14 and WILEY 09 mass spectral library. Peak area percentage indicates the relative concentration of each compound in the total ion chromatogram.
